# Dietary Total Antioxidant Capacity and Dietary Polyphenol Intake and Prevalence of Metabolic Syndrome in Polish Adults: A Nationwide Study

**DOI:** 10.1155/2018/7487816

**Published:** 2018-03-26

**Authors:** Małgorzata Elżbieta Zujko, Anna Waśkiewicz, Anna Maria Witkowska, Danuta Szcześniewska, Tomasz Zdrojewski, Krystyna Kozakiewicz, Wojciech Drygas

**Affiliations:** ^1^Department of Food Biotechnology, Medical University of Bialystok, Bialystok, Poland; ^2^Department of Epidemiology, Cardiovascular Disease Prevention and Health Promotion, Institute of Cardiology, Warsaw, Poland; ^3^Department of Prevention and Education and Department of Hypertension and Diabetology, Medical University of Gdansk, Gdansk, Poland; ^4^3rd Department of Cardiology, Upper Silesian Centre of Cardiology, Medical University of Silesia, Katowice, Poland; ^5^Department of Social and Preventive Medicine, Medical University of Lodz, Lodz, Poland

## Abstract

Specific classes and subclasses of polyphenols have been studied for their potential effects on noncommunicable diseases, but studies on association between dietary polyphenol intake (DPI) and dietary total antioxidant capacity (DTAC) and MetS (metabolic syndrome) are scarce. Therefore, the aim of this study was to determine associations between DTAC and DPI and the prevalence of MetS and its components in the Polish adult population. Subjects (5690) were participants of the Polish National Multicentre Health Examination Survey (WOBASZ II study) performed in 2013-2014. MetS was defined according to the International Diabetes Federation (IDF) and the American Heart Association/National Heart, Lung, and Blood Institute (AHA/NHLBI) criteria. Daily food consumption was assessed by 24-hour dietary recall. DTAC and DPI were evaluated using the data of food consumption and antioxidant potential of foods, measured by FRAP (ferric reducing antioxidant potential) method, and total polyphenol content in foods, measured by Folin-Ciocalteu assay. Logistic regression models were used to assess the relationship between DTAC and DPI and MetS and its components. Crude, age-adjusted, and multivariable-adjusted models were performed. This study demonstrated that in Polish women, high DPI and high DTAC were significantly associated with a reduced odds ratio for the prevalence of MetS components, such as elevated blood pressure and diabetes. In contrast, in men, high DPI and high DTAC did not have the potential to alleviate MetS components.

## 1. Introduction

Metabolic syndrome (MetS) is a clustering of risk factors, such as central obesity, elevated fasting glucose, elevated triglycerides, reduced high-density lipoprotein cholesterol, and elevated blood pressure, that together culminate in the increased risk of diabetes mellitus type 2 (DM2) and cardiovascular disease (CVD). The first formalized definition of MetS was proposed in 1998 by the World Health Organization (WHO) [1]. Over the past years, various diagnostic criteria have been presented by different organizations. Most recently, these have come from the International Diabetes Federation (IDF) and the American Heart Association/National Heart, Lung, and Blood Institute (AHA/NHLBI) [2].

The prevalence of MetS varies widely across population and depends on several factors, such as age, gender, socioeconomic status, education level, and lifestyle [3–5]. MetS is diagnosed in a third of the US population and a quarter of the European population [6, 7]. A consequence of metabolic syndrome is CVD and DM2, which are the leading public health problems with high socioeconomic cost. Currently, the global cost of diabetes is $825 billion dollars per year [8], while CVD costs US $555 billion per year [9] and €210 billion per year [10].

Several studies have reported that oxidative stress, caused by the imbalance between prooxidants and antioxidants in the living organism system, plays an important role in the prevalence of MetS [11, 12]. The overproduction of reactive oxygen species may impair insulin signalling pathways and lead to endothelial damage. This causes insulin resistance and promotes acceleration of the atherogenic process [13]. Moreover, oxidative stress and endothelial dysfunction are known to be associated with inflammation and can contribute to hypertension [14].

Modification of lifestyle, including healthy nutrition, is the primary approach for MetS prevention [15]. It was found that the dietary total antioxidant capacity (DTAC), which may increase after dietary polyphenol intake (DPI), has a significant influence on the serum total antioxidant status [16]. Some studies indicated that higher consumption of specific classes of polyphenols [17, 18] and DPI [18] and higher DTAC [19–21] have favourable effects on metabolic disorders in different population. Predominant sources of the dietary polyphenols in Polish population are tea, coffee, and apples [22, 23].

The objective of this study was to determine the associations between DTAC and DPI and the prevalence of MetS and its specific components in Polish adult population. To our knowledge, this is the first attempt to estimate the relationships between DTAC and MetS in a cross-sectional Polish study.

## 2. Material and Methods

### 2.1. Study Population

Subjects were participants of the Polish National Multicentre Health Examination Survey (WOBASZ II study) performed in 2013-2014, which was a cross-sectional study aimed at investigating the determinants of chronic noncommunicable diseases in a random sample of Polish residents aged 20 years or older. The project was conducted by the Institute of Cardiology in Warsaw in cooperation with 5 medical universities in Poland. The randomization had two stages. In each of 16 voivodeships, 6 communities were drawn, including 2 small (up to 8000 inhabitants), 2 medium (8000–40000 inhabitants), and 2 large (over 40000 inhabitants) ones, plus province capitals (unless it was selected as one of the communes). Thereafter, 70 men and 70 women were drawn from each community. Altogether, 15120 subjects (including 1557 respondents who were unavailable) of both genders were drawn from the Department of State Registry database, the Ministry of Internal Affairs (PESEL register). A total of 6170 respondents participated in the study, which was 45.5% of the total sample. Whereas, dietary recalls were obtained from 5690 people (2554 men and 3136 women). The aims and methods of the WOBASZ II study have been described in detail elsewhere [24, 25]. The participants provided written informed consent, and the study protocol was approved by the Bioethics Committee of the National Institute of Cardiology (number 1344).

### 2.2. Demographic and Lifestyle Information

Sociodemographic and lifestyle characteristics such as age, gender, educational level, occupational activity, physical activity, smoking habits, and alcohol intake were collected from self-reported standardized questionnaires. Educational level was classified as under middle (primary, vocational, and partial secondary education), middle (secondary and partial academic education), and high (licentiate or university education). Physical activity at leisure for at least 30 min a day was assessed as low (once a week or less), middle (2-3 times a week), and high level (≥4 times a week). Physical activity assessment was described in detail in a previous substudy [26]. Smoking status was categorised as smoking at least one cigarette a day. Alcohol intakes were calculated as gram pure ethanol a day.

### 2.3. Clinical Measurements

The measurements of body mass, height, waist circumference, and blood pressure were performed by trained nurses, using standardized procedures. Body weight was measured without footwear and without top garments, to the nearest 0.1 kg. Height was measured in the standing position without footwear, to the nearest 0.5 cm. Waist circumference was measured at the level of the umbilicus, using a measuring tape, to the nearest 0.5 cm. Body mass index (BMI) was calculated as body mass in kilograms divided by squared height in meters. A BMI of 18.5–24.9 kg/m^2^ was defined as normal body mass, a BMI of 25.0–29.9 kg/m^2^ was classified as overweight, and a BMI of over 30.0 kg/m^2^ was determined as obesity. Blood pressure was measured three times on the right arm, in a sitting position, at one-minute intervals, using automatic devices AND UA-631, approved by AAMI (Association for the Advancement of Medical Instrumentation). The average value from the second and the third measurements was used for the analysis.

Determinations of fasting glucose and blood lipids were carried out at a single location, Diagnostyka Central Laboratory at the Institute of Cardiology in Warsaw, which holds a CDC certificate (the Centre for Disease Control, Lipid Standardization Program, Atlanta, USA) and the European certificate RIQAS (Random International Quality Assessment Scheme). Measurements of fasting glucose and blood lipids were performed using enzymatic-colorimetric methods on the analyzer Cobas 6000, manufactured by Roche.

### 2.4. MetS Criteria

MetS criteria were adopted according to the International Diabetes Federation (IDF) and the American Heart Association/National Heart, Lung, and Blood Institute (AHA/NHLBI) definition [2]. MetS was diagnosed when at least three of five risk factors have been identified: (1) elevated waist circumference (for European population: ≥94 cm for men and ≥ 80 cm for women); (2) elevated triglycerides (≥150 mg/dl–1.7 mmol/l); (3) reduced HDL cholesterol (<40 mg/dl–1.0 mmol/l for men and <50–1.3 mmol/l for women); (4) elevated blood pressure (systolic ≥ 130 and/or diastolic ≥ 85 mmHg) or hypertension in interview; and (5) elevated fasting glucose (≥100 mg/dl–5.6 mmol/l) or diabetes in interview.

### 2.5. Dietary Assessment

Dietary assessment was performed by qualified interviewers, using a single 24-hour dietary recalls. Food portion sizes were estimated using an album with photographs of the most consumed food products established by the National Food and Nutrition Institute, Warsaw, Poland. It was found that 367 of the variety of foods were sources of polyphenol intakes among the whole population of study. These products were grouped in 5 food categories, namely, beverages (alcoholic and nonalcoholic), cereals, fruit, vegetables, and other food products (e.g., chocolates, nuts, and seeds). Individual components of complex dishes were extracted using dish recipes obtained from the Polish Food Composition Tables [27].

### 2.6. Estimation of DTAC and DPI

DTAC and DPI were determined by multiplying the daily consumption of individual food items by antioxidant capacity of foods and polyphenol contents in these food items. Antioxidant potential of foods, measured by FRAP method (ferric reducing antioxidant potential), was mostly taken from Polish databases [28, 29], and some missing values were complemented with another database [30]. Data on the total polyphenol content in foods, measured by Folin-Ciocalteu assay, were obtained from Polish databases [28, 29], as well as the online Phenol-Explorer database [31].

### 2.7. Statistical Analysis

Statistical analyses were performed using a Statistical Analysis System (SAS) software, version 9.2. 2 (SAS Institute Inc., Cary, NC). Continuous variables were presented as means and standard deviations (SD) and categorical variables as counts and percentages. Kruskal-Wallis test was used for comparison of continuous variables and chi-square test for categorical variables across categories of DPI and DTAC. Logistic regression models were used to assess the relationship between DPI and DTAC and MetS and its components. Crude, age-adjusted, and multivariable-adjusted models were performed. The results were presented as odds ratios (ORs) and 95% confidence intervals (CIs) of the associations between MetS and its specific components and tertiles (T1–T3) of DPI and DTAC. The level of significance for two-sided tests was considered to be at *p* < 0.05.

## 3. Results

Demographic and lifestyle characteristics of the 5690 participants (2554 men and 3136 women), stratified by occurrence of MetS, are given in Table 1. The mean age of the studied population was 50.08 ± 16.44 y, and the mean BMI was 27.17 ± 5.19 kg/m^2^. It was found that MetS criteria have been met by 36% of participants (39% men and 33% women). Subjects of older age, higher BMI, low education level, low leisure time physical activity, lack of employment, and high alcohol intake were more likely to have MetS.

Anthropometric characteristics and biomarkers of metabolic syndrome by tertiles of DPI and DTAC are presented in Tables 2 and 3, respectively. Higher DPI and higher DTAC were significantly associated with lower fasting glucose and lower blood pressure, but only in women. There was no significant association in men.

The odds ratios for MetS and its specific components by tertiles of DPI and DTAC were evaluated individually for men and women, using multiple logistic regression analysis (Tables 4 and 5, resp.). Three models were tested: model 1—crude data, model 2—data adjusted for age, and model 3—data adjusted for age, BMI, educational level, leisure time physical activity, smoking, and alcohol intake. The first tertile (T1) in each model was adopted as a reference. For both genders, the associations between DPI and MetS as well as between DTAC and MetS were not significant after adjustment for multiple covariates (model 3). In men, higher DPI and higher DTAC were associated with reduced odds of diabetes between T3 and T1 (OR = 0.673, 95% CI = 0.495–0.917 and OR = 0.684, 95% CI = 0.506–0.925, resp.), but only when the crude data were taken into consideration (model 1). In a single model (model 2) and in a multiple adjustment model (model 3), associations were not statistically significant. Ambiguous results were found for DPI and HDL-cholesterol. In men, higher DPI was significantly associated with lower HDL cholesterol (T3 versus T1; model 3; OR = 1.410, 95% CI = 1.080–1.842); such association was not found for women.

In women, individuals with higher DPI had lower blood pressure (Table 4). When the analysis was stratified by multiple covariates (model 3), the odds of elevated blood pressure were 22.3% lower in T2 (OR = 0.777, 95% CI = 0.620–0.975) and 30.6% lower in T3 (OR = 0.694, 95% CI = 0.556–0.867) in comparison to T1. Higher DTAC was associated with 22.8% lower odds of elevated blood pressure between T3 and T1 in model 3 (OR = 0.772, 95% CI = 0.619–0.963) (Table 5). In addition, higher DPI and DTAC in women were significantly associated with reduced odds of elevated blood glucose (FG ≥ 100 mg/dl (5.6 mmol/dl) or diabetes in interview), but only in model 1. After adjustment for confounding factors (model 3), significant associations were found only for diabetes (FG ≥ 126 mg/dl (7 mmol/l) or diabetes in interview) between T2 and T1. In women, higher DPI was associated with 28.2% lower odds of diabetes (OR = 0.718, 95% CI = 0.520–0.992), whereas higher DTAC was associated with 27.9% lower odds of diabetes (OR = 0.721, 95% CI = 0.522–0.997).

## 4. Discussion

In the present study, we evaluated the relationship between DPI and DTAC and MetS and its specific components in Polish representative population of both genders. Inverse associations were evident in women between high DPI, high DTAC, and MetS components, such as elevated blood pressure and diabetes (in a middle tertile). In contrast, in men, high DPI and high DTAC did not have the potential to alleviate MetS components. Moreover, men who consumed more polyphenol had lower HDL-cholesterol. These differences between genders can be explained by different nutrition habits of both genders and different cut-off points set as criteria for HDL-cholesterol.

To date, only a limited number of epidemiological studies evaluated the association between DPI and MetS and its components. Our results regarding this association between higher DPI and lower risk of diabetes and hypertension in women are in accordance with those found in the HAPIEE (Health, Alcohol and Psychosocial factors In Eastern Europe cohort) study [32, 33]. Similarly, an observational cohort analysis of the nondiabetic participants in the PREDIMED (PREvencion con DIeta MEDiterranea) trial showed that higher DPI was associated with a reduced risk of diabetes in elderly persons at high risk of cardiovascular disease [34]. Moreover, another HAPIEE study indicated that high DPI was negatively associated with MetS components, such as waist circumference, blood pressure, HDL-cholesterol, and triglycerides in women and fasting plasma glucose in both genders [18].

Most of the current epidemiological studies have shown that higher consumption of polyphenols have been related to decreased risk of MetS components, such as diabetes [17, 18, 32, 34–37], hypertension [17, 18, 33, 38–41], central obesity [17, 18], dyslipidemia [17, 18, 42, 43], and incidence of cardiovascular events [44], although knowledge on this subject is still scarce and the results are inconsistent. Some studies indicate on the positive impact of dietary total polyphenol intake on lower prevalence of MetS and its components [18, 32–34, 42], while others show these associations only for selected subclasses of polyphenols [17, 35, 37–40]. Our study added to the current knowledge regarding positive results of dietary total polyphenol intake on lower blood pressure and occurrence of diabetes in Polish women in a cross-sectional study. It should be pointed that polyphenols are characterized by low bioavailability and their maximal plasma concentrations are reached within the first two hours after ingestion and fall to baseline levels within 8–12 hours. Therefore, the protective effects of polyphenols on MetS may be reached through a long-term regular, daily consumption [45].

Polyphenol-rich foods, such as fruits and vegetables, may exert antioxidant activity, which are the sum of the effects of polyphenols and antioxidant vitamins [16]. In our study, after adjustment for confounding factors, DTAC was significantly associated with lower odds of elevated blood pressure and diabetes (in a middle tertile) in women. However, population studies that examine the relationship between the DTAC and MetS and its components are limited. Similarly to our findings, the results of the EPIC (European Prospective Investigation into Cancer and Nutrition) study showed that the DTAC may play an important role in reducing the risk of diabetes in women [46]. Whereas, the longitudinal TLGS (Tehran Lipid and Glucose Study) showed that higher DTAC was positively associated with lower occurrence of MetS, abdominal obesity, and hypertension [19]. Findings of the Hertfordshire Cohort Study suggested that DTAC may have important protective effects on glucose tolerance, especially in older obese women [47]. A case-control study showed that higher dietary total antioxidant capacity is inversely related to prediabetes morbidity [48].

In our study, the prevalence of MetS was identified in 36% of Polish adults, with higher prevalence in men than in women. It was found that the prevalence of MetS in Polish population was increased from 25% to 36% between 2003–2005 and 2013-2014 [7]. Our findings correspond with the results of the NHANES (National Health and Nutrition Examination Survey) study, which showed the increase of MetS among US adults (from 29% to 34% between 1988–1994 and 1999–2006, with a greater increase of MetS in women) [49]. In our study, MetS was dependent on various factors, such as gender, age, BMI, education level, leisure time physical activity, employment, and alcohol intake, which is in accordance with other studies [3–7].

Our study has some limitations. The participation rate in the WOBASZ II study was rather low (45.5%), but typical for cross-sectional studies. This problem was discussed in a previous publication [24]. Next, food intakes were estimated with the single 24-hour dietary recall method that does not reflect habitual or long-term food consumption. However, the 24-hour recall is a common method, which is useful to estimate mean food intakes in large groups of participants. Moreover, this study might have underestimated intakes of some polyphenols and the dietary antioxidant potential of some foods, which have been omitted in the dietary interview or were not included in the polyphenol and antioxidant databases. It was previously found that polyphenol intake estimated according to various databases may substantially differ [50]. Finally, polyphenols in foods and dietary antioxidant capacity can interact with other constituents of foods, which may contribute to the association with prevalence of metabolic disorders. Despite such limitations, this study included a large randomly selected sample of Polish adult population, and clinical measurements and demographic and lifestyle information were based on the standardized procedures.

## 5. Conclusion

This study demonstrated that in Polish women, high DPI and high DTAC were significantly associated with a reduced odds ratio for the prevalence of MetS components, such as elevated blood pressure and diabetes. In contrast, in men, high DPI and high DTAC did not have the potential to alleviate MetS components.

## Figures and Tables

**Table 1:** General characteristics of studied population (*N* = 5690) according to prevalence of metabolic syndrome.

	Total	Metabolic syndrome		*p*
		No	Yes	
Gender, *N* (%)	5690 (100)	3662 (64.36)	2028 (35.64)	<0.0001
Men	2554 (44.89)	1554 (60.85)	1000 (39.15)	
Women	3136 (55.11)	2108 (67.22)	1028 (32.78)	
Age (years), mean ± SD	50.08 **±** 16.44	46.11 **±** 16.32	57.25 ± 14.04	<0.0001
Age, *N* (%)				<0.0001
20–39	1724 (30.30)	1485 (86.14)	239 (13.86)	
40–59	2202 (38.70)	1346 (61.13)	856 (38.87)	
>60	1764 (31.00)	831 (47.11)	933 (52.89)	
BMI (kg/m^2^), mean ± SD	27.17 **±** 5.19	25.44 **±** 4.59	30.24 **±** 4.76	<0.0001
BMI, *N* (%)				<0.0001
<18.5 kg/m^2^	91 (1.61)	91 (100.00)	0 (0.00)	
18.5–24.9 kg/m^2^	1985 (34.91)	1758 (88.58)	227 (11.42)	
25–29.9 kg/m^2^	2120 (37.25)	1283 (60.53)	837 (39.47)	
>30 kg/m^2^	1494 (26.23)	505 (33.78)	989 (66.22)	
Educational level, *N* (%)				<0.0001
Under middle	2274 (39.96)	1235 (54.29)	1039 (45.71)	
Middle	2140 (37.62)	1425 (66.60)	715 (33.40)	
High	1276 (22.42)	1002 (78.49)	274 (21.51)	
Leisure time physical activity, *N* (%)				<0.0001
Low level	2614 (45.94)	1601 (61.23)	1013 (38.77)	
Middle level	1478 (25.98)	1035 (70.06)	443 (29.94)	
High level	1598 (28.08)	1028 (64.32)	570 (35.68)	
Currently working, *N* (%)				<0.0001
Yes	2980 (52.37)	2127 (71.39)	853 (28.61)	
No	2710 (47.63)	1534 (56.59)	1176 (43.41)	
Currently smoking, *N* (%)				0.0575
Yes	1647 (28.95)	1049 (63.69)	598 (36.31)	
No	4043 (71.05)	2411 (59.64)	1632 (40.36)	
Alcohol intake (g ethanol/day), mean ± SD	5.74 **±** 12.25	5.50 **±** 11.80	6.18 **±** 13.01	0.0368
Energy intake (kcal/day), mean ± SD	1965.0 **±** 847.9	1975.0 **±** 831.8	1946.9 **±** 876.1	0.0839
DPI (mg/day), mean ± SD	2025.0 **±** 892.0	2036.0 **±** 886.5	2005.2 **±** 901.9	0.1251
DTAC (mmol/day), mean ± SD	12.31 **±** 7.37	12.34 **±** 6.86	12.27 **±** 8.22	0.2567

*N*: number; BMI: body mass index; DTAC: dietary total antioxidant capacity; DPI: dietary polyphenol intake.

**Table 2:** Anthropometric characteristics and biomarkers of metabolic syndrome by tertiles of DPI and by gender, expressed as mean ± SD (*N* = 5690).

	Tertile of DPI, men (*N* = 2554)			*p*	Tertile of DPI, women (*N* = 3136)			*p*
	T1 mean ± SD (range) 1153 ± 323 (174–1599)	T2 mean ± SD (range) 1969 ± 211 (1600–2346)	T3 mean ± SD (range) 3092 ± 785 (2347–9048)		T1 mean ± SD (range) 1152 ± 313 mg (140–1587)	T2 mean ± SD (range) 1904 ± 186 mg (1588–2239)	T3 mean ± SD (range) 2904 ± 691 mg (2240–8793)	
BMI (kg/m^2^)	27.30 ± 4.44	27.42 ± 4.67	27.53 ± 4.53	0.7198	27.18 ± 5.64	27.00 ± 5.65	26.71 ± 5.66	0.0946
WC (cm)	97.31 ± 12.16	97.39 ± 13.04	97.00 ± 12.04	0.8493	88.34 ± 13.79	88.48 ± 14.06	87.18 ± 13.46	0.0566
TC (mmol/l)	5.22 ± 1.33	5.17 ± 1.27	5.23 ± 1.40	0.7300	5.18 ± 1.24	5.17 ± 1.19	5.23 ± 1.23	0.3279
LDL-C (mmol/l)	3.19 ± 1.03	3.17 ± 1.04	3.21 ± 1.04	0.7121	3.10 ± 1.04	3.08 ± 0.98	3.16 ± 1.03	0.1525
HDL-C (mmol/l)	1.33 ± 0.40	1.32 ± 0.43	1.33 ± 0.46	0.6841	1.53 ± 0.42	1.53 ± 0.41	1.53 ± 0.43	0.8516
TG (mmol/l)	1.80 ± 1.92	1.74 ± 1.53	1.76 ± 2.21	0.4551	1.38 ± 0.81	1.37 ± 0.81	1.32 ± 0.75	0.3001
FG (mmol/l)	5.69 ± 1.59	5.62 ± 1.31	5.63 ± 1.85	0.9001	5.56 ± 1.69	5.34 ± 1.20	5.26 ± 0.96	0.0006
SBP (mmHg)	135.5 ± 19.1	134.3 ± 18.0	133.5 ± 17.4	0.1357	130.1 ± 20.6	127.0 ± 19.3	125.8 ± 19.0	<0.0001
DBP (mmHg)	81.8 ± 11.5	81.3 ± 11.1	81.5 ± 10.1	0.8439	79.9 ± 10.6	78.7 ± 10.7	79.0 ± 10.6	0.0242

BMI: body mass index; WC: waist circumference; TC: total cholesterol; LDL-C: low-density lipoprotein cholesterol; HDL-C: high-density lipoprotein cholesterol; TG: triglycerides; FG: fasting glucose; SBP: systolic blood pressure; DBP: diastolic blood pressure.

**Table 3:** Anthropometric characteristics and biomarkers of metabolic syndrome by tertiles of DTAC and by gender, expressed as mean ± SD (*N* = 5690).

	Tertile of DTAC, men (*N* = 2554)			*p*	Tertile of DTAC, women (*N* = 3136)			*p*
	T1 mean ± SD (range) 5.98 ± 2.02 (0.47–8.83)	T2 mean ± SD (range) 11.24 ± 1.42 (8.84–13.84)	T3 mean ± SD (range) 19.89 ± 8.00 (13.84–95.69)		T1 mean ± SD (range) 6.28 ± 2.03 (0.32–9.18)	T2 mean ± SD (range) 11.39 ± 1.28 (9.19–13.73)	T3 mean ± SD (range) 19.12 ± 8.31 (13.74–191.82)	
BMI (kg/m^2^)	27.29 ± 4.51	27.43 ± 4.59	27.53 ± 4.54	0.6124	27.15 ± 5.71	26.99 ± 5.77	26.75 ± 5.48	0.3117
WC (cm)	97.41 ± 12.37	96.92 ± 12.89	97.37 ± 11.99	0.4135	88.30 ± 14.09	88.37 ± 13.96	87.32 ± 13.26	0.1440
TC (mmol/l)	5.17 ± 1.34	5.19 ± 1.29	5.25 ± 1.38	0.2944	5.17 ± 1.24	5.20 ± 1.24	5.21 ± 1.18	0.4813
LDL-C (mmol/l)	3.14 ± 1.00	3.18 ± 1.06	3.25 ± 1.04	0.2028	3.09 ± 1.04	3.12 ± 1.01	3.13 ± 1.01	0.4652
HDL-C (mmol/l)	1.32 ± 0.39	1.32 ± 0.42	1.34 ± 0.47	0.6955	1.52 ± 0.41	1.54 ± 0.43	1.54 ± 0.42	0.5762
TG (mmol/l)	1.81 ± 1.99	1.80 ± 2.19	1.68 ± 1.48	0.5592	1.38 ± 0.87	1.35 ± 0.73	1.34 ± 0.76	0.5926
FG (mmol/l)	5.69 ± 1.50	5.63 ± 1.81	5.62 ± 1.47	0.4232	5.50 ± 1.60	5.39 ± 1.27	5.35 ± 1.27	0.0080
SBP (mmHg)	135.4 ± 19.0	134.3 ± 18.5	133.6 ± 17.0	0.1637	129.3 ± 20.5	127.8 ± 19.8	125.8 ± 18.7	0.0008
DBP (mmHg)	81.6 ± 11.5	81.4 ± 11.1	81.5 ± 10.2	0.8380	79.3 ± 10.8	79.3 ± 10.7	79.0 ± 10.4	0.8367

BMI: body mass index; WC: waist circumference; TC: total cholesterol; LDL-C: low-density lipoprotein cholesterol; HDL-C: high-density lipoprotein cholesterol; TG: triglycerides; FG: fasting glucose; SBP: systolic blood pressure; DBP: diastolic blood pressure.

**Table 4:** Odds ratios (OR) (95% CI (confidence interval)) for metabolic syndrome and its specific components by tertiles of DPI and by gender (*N* = 5690).

Characteristic	Tertile of DPI, men (*N* = 2554)			Tertile of DPI, women (*N* = 3136)		
	T1 (ref.) 174–1599	T2 1600–2346	T3 2347–9048	T1 (ref.) 140–1587	T2 1588–2239	T3 2240–8793
Metabolic syndrome						
Crude OR (95% CI)	1	0.990 (0.815–1.204)	1.074 (0.885–1.305)	1	0.813 (0.678–0.976)	0.820 (0.683–0.983)
Adjusted OR (95% CI)^1^	1	1.024 (0.836–1.254)	1.155 (0.944–1.412)	1	0.910 (0.745–1.111)	1.025 (0.840–1.250)
Adjusted OR (95% CI)^2^	1	0.908 (0.720–1.144)	1.044 (0.830–1.312)	1	0.861 (0.688–1.078)	0.952 (0.761–1.191)
WC ≥ 94 cm (men), ≥80 cm (women)						
Crude OR (95% CI)	1	0.992 (0.815–1.208)	1.065 (0.875–1.298)	1	0.908 (0.750–1.098)	0.883 (0.730–1.068)
Adjusted OR (95% CI)^1^	1	1.010 (0.822–1.241)	1.113 (0.906–1.367)	1	0.994 (0.806–1.226)	0.987 (0.802–1.215)
Adjusted OR (95% CI)^2^	1	0.981 (0.737–1.307)	1.079 (0.812–1.432)	1	1.033 (0.779–1.370)	1.080 (0.818–1.426)
TG ≥ 150 mg/dl (1.7 mmol/l)						
Crude OR (95% CI)	1	0.889 (0.725–1.088)	0.939 (0.768–1.148)	1	0.992 (0.805–1.221)	0.867 (0.702–1.071)
Adjusted OR (95% CI)^1^	1	0.890 (0.726–1.090)	0.941 (0.769–1.150)	1	1.055 (0.853–1.306)	0.964 (0.776–1.197)
Adjusted OR (95% CI)^2^	1	0.859 (0.691–1.066)	0.899 (0.726–1.115)	1	1.066 (0.849–1.338)	0.937 (0.743–1.182)
Low HDL-C < 40 mg/dl (1.0 mmol/l) (men), <50 mg/dl (1.3 mmol/l) (women)						
Crude OR (95% CI)	1	1.102 (0.851–1.429)	1.425 (1.111–1.828)	1	0.889 (0.734–1.077)	0.926 (0.765–1.120)
Adjusted OR (95% CI)^1^	1	1.106 (0.853–1.433)	1.433 (1.117–1.840)	1	0.921 (0.759–1.119)	0.985 (0.812–1.195)
Adjusted OR (95% CI)^2^	1	1.080 (0.818–1.426)	1.410 (1.080–1.842)	1	0.925 (0.751–1.140)	0.939 (0.762–1.157)
SBP ≥ 130 mmHg or DBP ≥ 85 mmHg or hypertension in interview						
Crude OR (95% CI)	1	0.904 (0.737–1.109)	0.855 (0.698–1.047)	1	0.743 (0.625–0.884)	0.638 (0.536–0.759)
Adjusted OR (95% CI)^1^	1	0.922 (0.741–1.146)	0.869 (0.701–1.077)	1	0.782 (0.634–0.965)	0.703 (0.572–0.863)
Adjusted OR (95% CI)^2^	1	0.956 (0.759–1.203)	0.887 (0.706–1.113)	1	0.777 (0.620–0.975)	0.694 (0.556–0.867)
FG ≥ 100 mg/dl (5.6 mmol/dl) or diabetes in interview						
Crude OR (95% CI)	1	1.049 (0.859–1.281)	1.023 (0.838–1.249)	1	0.766 (0.629–0.934)	0.692 (0.566–0.845)
Adjusted OR (95% CI)^1^	1	1.106 (0.894–1.370)	1.125 (0.910–1.390)	1	0.845 (0.681–1.049)	0.854 (0.687–1.061)
Adjusted OR (95% CI)^2^	1	1.048 (0.839–1.310)	1.057 (0.848–1.319)	1	0.821 (0.652–1.034)	0.883 (0.700–1.130)
FG ≥ 126 mg/dl (7 mmol/l) or diabetes in interview						
Crude OR (95% CI)	1	0.922 (0.691–1.231)	0.673 (0.495–0.917)	1	0.649 (0.488–0.862)	0.534 (0.397–0.720)
Adjusted OR (95% CI)^1^	1	1.023 (0.752–1.392)	0.829 (0.598–1.150)	1	0.752 (0.556–1.017)	0.739 (0.538–1.014)
Adjusted OR (95% CI)^2^	1	0.964 (0.697–1.333)	0.797 (0.566–1.121)	1	0.718 (0.520–0.992)	0.780 (0.558–1.091)

WC: waist circumference; TG: triglycerides; HDL-C: high-density lipoprotein cholesterol; SBP: systolic blood pressure; DBP: diastolic blood pressure; FG: fasting glucose. ^1^Analysis adjusted for age; ^2^analysis adjusted for age, BMI, educational level, leisure time physical activity, smoking, and alcohol intake.

**Table 5:** Odds ratios (OR) (95% CI (confidence interval)) for metabolic syndrome and its specific components by tertiles of DTAC and by gender (*N* = 5690).

	Tertile of DTAC, men (*N* = 2554)			Tertile of DTAC, women (*N* = 3136)		
	T1 (ref.) 0.47–8.83	T2 8.84–13.84	T3 13.84–95.69	T1 (ref.) 0.32–9.18	T2 9.19–13.73	T3 13.74–191.82
Metabolic syndrome						
Crude OR (95% CI)	1	1.010 (0.831–1.227)	1.038 (0.855–1.261)	1	0.946 (0.788–1.134)	0.900 (0.750–1.080)
Adjusted OR (95% CI)^1^	1	1.057 (0.863–1.294)	1.099 (0.899–1.345)	1	1.005 (0.823–1.227)	1.126 (0.922–1.376)
Adjusted OR (95% CI)^2^	1	0.985 (0.782–1.242)	1.010 (0.803–1.270)	1	1.019 (0.815–1.274)	1.043 (0.832–1.306)
WC ≥ 94 cm (men), ≥80 cm (women)						
Crude OR (95% CI)	1	0.911 (0.749–1.109)	1.088 (0.893–1.326)	1	1.013 (0.837–1.225)	0.946 (0.783–1.143)
Adjusted OR (95% CI)^1^	1	0.934 (0.760–1.147)	1.124 (0.915–1.381)	1	1.021 (0.828–1.259)	1.048 (0.852–1.288)
Adjusted OR (95% CI)^2^	1	0.798 (0.598–1.066)	1.020 (0.766–1.356)	1	1.163 (0.877–1.543)	1.065 (0.808–1.403)
TG ≥ 150 mg/dl (1.7 mmol/l)						
Crude OR (95% CI)	1	1.083 (0.885–1.326)	0.962 (0.786–1.179)	1	0.994 (0.806–1.226)	0.939 (0.761–1.159)
Adjusted OR (95% CI)^1^	1	1.085 (0.886–1.328)	0.964 (0.787–1.181)	1	1.020 (0.823–1.263)	1.035 (0.834–1.284)
Adjusted OR (95% CI)^2^	1	1.037 (0.836–1.288)	0.941 (0.759–1.169)	1	1.016 (0.809–1.276)	0.999 (0.793–1.258)
Low HDL-C < 40 mg/dl (1.0 mmol/l) (men), <50 mg/dl (1.3 mmol/l) (women)						
Crude OR (95% CI)	1	1.111 (0.860–1.435)	1.269 (0.988–1.629)	1	0.965 (0.797–1.169)	0.942 (0.778–1.140)
Adjusted OR (95% CI)^1^	1	1.115 (0.863–1.440)	1.274 (0.992–1.636)	1	0.977 (0.805–1.186)	0.994 (0.819–1.207)
Adjusted OR (95% CI)^2^	1	1.149 (0.873–1.513)	1.296 (0.991–1.696)	1	1.059 (0.861–1.304)	0.939 (0.760–1.159)
SBP ≥ 130 mm Hg or DBP ≥ 85 mm Hg or hypertension in interview						
Crude OR (95% CI)	1	0.815 (0.664–1.000)	0.817 (0.666–1.002)	1	0.876 (0.737–1.041)	0.709 (0.596–0.843)
Adjusted OR (95% CI)^1^	1	0.820 (0.660–1.020)	0.806 (0.649–1.001)	1	0.878 (0.712–1.083)	0.789 (0.643–0.969)
Adjusted OR (95% CI)^2^	1	0.816 (0.648–1.028)	0.812 (0.646–1.021)	1	0.891 (0.711–1.117)	0.772 (0.619–0.963)
FG ≥ 100 mg/dl (5.6 mmol/dl) or diabetes in interview						
Crude OR (95% CI)	1	0.965 (0.790–1.178)	1.016 (0.833–1.240)	1	0.854 (0.700–1.040)	0.737 (0.603–0.902)
Adjusted OR (95% CI)^1^	1	1.018 (0.822–1.261)	1.096 (0.887–1.354)	1	0.888 (0.716–1.102)	0.899 (0.723–1.118)
Adjusted OR (95% CI)^2^	1	0.991 (0.792–1.239)	1.060 (0.850–1.321)	1	0.859 (0.683–1.082)	0.907 (0.718–1.146)
FG ≥ 126 mg/dl (7 mmol/l) or diabetes in interview						
Crude OR (95% CI)	1	0.793 (0.592–1.062)	0.684 (0.506–0.925)	1	0.701 (0.527–0.934)	0.619 (0.461–0.830)
Adjusted OR (95% CI)^1^	1	0.889 (0.651–1.213)	0.821 (0.596–1.131)	1	0.754 (0.556–1.022)	0.851 (0.621–1.166)
Adjusted OR (95% CI)^2^	1	0.896 (0.645–1.245)	0.871 (0.623–1.216)	1	0.721 (0.522–0.997)	0.873 (0.625–1.220)

WC: waist circumference; TG: triglycerides; HDL-C: high-density lipoprotein cholesterol; SBP: systolic blood pressure; DBP: diastolic blood pressure; FG: fasting glucose. ^1^Analysis adjusted for age; ^2^analysis adjusted for age, BMI, educational level, leisure time physical activity, smoking, and alcohol intake.
